# Chicago Society of Ophthalmology and Otology; Meeting of February 8th

**Published:** 1887-06

**Authors:** 


### THE CHICAGO SOCIETY OF OPHTHALMOLOGY AND OTOLOGY.

*Meeting of February 8th.*—THE PRESIDENT, E. L. HOLMES, M. D., in the chair.

PROFESSOR W. F. COLEMAN read a paper on

### THE ADJUSTMENT OF GLASSES IN ERRORS OF REFRACTION.

in cases of simple myopic astigmatism of low degree, in which he said he is "inclined to think it would always be best for near vision to substitute a plus cylinder, with its axis at right angles to that of the minus cylinder found for distances."

He dissents from the view held by Doctor Culbertson as to the manner in which a plus cylinder will correct the ametropia in these cases of simple myopic astigmatism. Doctor Culbertson, in the *American Journal of Ophthalmology*, says: "By this minus glass for distance the myopia in proximal vision has been simply transferred from one to the other plane, and the foci of the two planes are not of the same length." Professor Coleman differs from that view, and cited several cases from his practice in which plus cylinders had been used in accordance with the plan reccommended, in which the results were reported as having been very satisfactory.

In discussing the paper, PROFESSOR E. J. GARDINER stated that he preferred to give the same glass for reading as for distance. He wished the eye to have the normal amount of accommodation, and did not care to give two kinds of glasses—one for distance: a minus cylinder; and one for reading: a plus cylinder. Professor B. Bettman had always prescribed a minus cylinder for reading. After Professor Coleman had mentioned to him the subject of the substitution of a plus cylinder for a minus one he had tried it in four cases, and in two of them the result had not been satisfactory.

PROFESSOR E. L. HOLMES said he had tried the substitution of a plus cylinder for the near-point, and had found the minus cylinder more satisfactory.

DR. H. GRADLE said he had always found a minus cylinder more satisfactory than a plus one. He had frequently noticed that the axis of the cylinder, determined with homatropine, differed from that determined when the accommodation had recovered, but that the patient always accepted the former in reading.

PROFESSOR GARDINER said he had frequently noticed the patient, in reading, change the axis of the distance cylinder.

PROFESSOR COLEMAN, in reply, said that, in his experience, patients with low degrees of myopic astigmatism did not care to wear glasses for distance.

PROFESSOR E. L. HOLMES presented for inspection a cataract glass, double convex cylindrical, axes at right angle and equivalent to + 12, D. spherical, ground by Meyrowitz Brothers, of New York. He stated that such lenses had been long in use by watch-makers and in the construction of certain optical instruments. His attention was called to the subject more than a year ago by a friend—a distinguished surgeon of New York—upon whom had been performed a successful operation for cataract. The usual glasses, if Professor Holmes remembered correctly, gave perfect satisfaction.

After a secondary operation for capsular remains, the patient found difficulty in adjusting spherical glasses. He then experimented with cylindrical lenses with great satisfaction.

There is reason to believe that this patient is one of the first that ever wore such a combination as being superior to spherical lenses. The patient, after visiting some of the European clinics, was led to believe that few, if any, of the teachers knew by practical experience that such cataract glasses were advantageous to some patients.

Experience must determine whether the slightly less spherical and chromatic aberration and the somewhat enlarged field of the cylindrical lens, such as was here presented, can be of special benefit to cataract patients, and those requiring strong, positive lenses.

PROFESSOR HOLMES also presented a small instrument for incising secondary capsular cataract, in cases in which iridectomy had been performed. The instrument is practically a very narrow Graefe's knife, bent on the flat about half an inch from the point—making a right angle with a square shoulder. The metal portion of the handle is round and small, not unlike that of a cataract needle. The handle of the instrument, in operating, is held in position as in making the corneal incision for extraction of cataract. The point is carried through the periphery of the cornea into the anterior chamber, the plane of the knife being in the plane of the perpendicular meridian of the globe. A slight rotation of the handle carries the cutting edge of the instrument through the opaque capsule. Dr. Holmes had used this instrument in three cases with great success. In a fourth case the very tough membrane was simply torn from its attachments and displaced.

The wound in the cornea produced by the instrument is somewhat larger than that made by a stop needle. There is less violence, however, to the tissues, than in cases in which it is necessary to make extensive motions with the handle of a stop-needle or of Knapp's knife-needle to insure rupture of the membranes. An instrument—right or left—is required for each eye.

PROFESSOR J. E. COLEMAN presented a paper in which he gave the results of his experience in the use of

THE GALVANIC CURRENT OF ELECTRICITY IN THE TREATMENT OF CERTAIN FORMS OF CATARACT

and classed the results into groups.

Group 1. "Cases in which there was no improvement." Group 2. "Cases that have been under observation from four to seven years, in which the improvement has been permanent." Group 3. "Cases in which there has been complete removal of short lines nebulæ." Group 4. "Recent cases in which there has been complete absorption of opacities." He said: If the cataract is due to the disturbance of nutrition or change in vascular supply, and in a large number of cases preceded by a marked disturbance of the general health, is it not fair to suppose that in those cases cited by Bull and others where there is arrested development, delayed maturing, or spontaneous absorption that the cause for the change may lie in the improvement in nutrition? That the remedy fails in certain percentage of the case, or in fact, all the cases under observation, may not be the fault of the remedy but in the selection of the cases to be treated *In all cases where the disease is progressive*, as indicated by the fat granules and nebula, where electricity is well borne, where the choroid and retina are not greatly degenerated, and where there are no complications of cirrhosis of kidneys or liver, diabetes or organic disease of heart or lungs, *improvement may be expected.* The cases in which improvement is not to be expected, are those in which vision has remained stationary for some time, and where there are structural changes in the retina and choroid.

*Meeting of February 12th.*—THE PRESIDENT, E. L. HOLMES, M.D., in the chair.

PROFESSOR E. L. HOTZ reported

A CASE OF PARTIAL TRICHIASIS RELIEVED BY STELLWAG'S METHOD OF REVERSING AND REPLANTING THE CILIARY BORDER, in which he said the feasibility of skin-grafting suggested to Professor Stellwag a new way of operating for trichiasis, which he announced in the *Allgemeine Wiener Medicinische Zeitschrift* in 1883. In the operation the border of the lid is split, making "an anterior and a posterior leaf, and a transverse incision parallel to the lid border and four to five millimeters from it, is made from canthus to canthus, but at each canthus the direction of this incision is so changed that there it joins the ends of the first cut by which the lid border has been split. In this way a long and narrow flap is obtained which contains all the eyelashes, this flap is completely dissected off; but instead of being thrown away, as in scalping, it is, after all bleeding of the wound has ceased, put back in its place again but with its edges reversed so that the originally upper edge is now the lower edge and *vice versa.* By this reversion of the flap the direction of the eyelashes is not only thoroughly changed so as to turn them away from the eyeball, but they are also removed a little distance from the edge of the lid by the interposition of a narrow strip of smooth skin, which serves as a sort of a substitute for the obliterated intermarginal border, and effectually prevents the eyelashes from ever again coming in contact with the cornea.

The operation had not always been successful in the hands of Professor Stellwag. If unsuccessful the hair bulbs may be destroyed and the eyelashes be lost, or the transplantation may be followed by the death of the transplanted part; in which case the operation would practically be the old operation of "scalping."

